# Oral Health Conditions in Patients with Parkinson’s Disease

**DOI:** 10.2188/jea.14.143

**Published:** 2005-03-18

**Authors:** Yoshimi Nakayama, Masakazu Washio, Mitsuru Mori

**Affiliations:** 1 Hokkaido Iwamizawa Public Health Center.; 2 Department of Public Health, Sapporo Medical University School of Medicine

**Keywords:** Oral Health, Parkinson Disease, Dental Care, Deglutition Disorders, Pneumonia, Aspiration

## Abstract

BACKGROUND: Oral health conditions and related factors of patients with Parkinson’s disease (PD) have not been well elucidated. The aim of the present study was to investigate oral health conditions and related factors which may influence oral health conditions among patients with PD.

METHODS: We compared oral health conditions and related factors between 104 PD patients and 191 inhibitants (controls) who received dental health check-ups in Hokkiado, Japan. The unconditional logistic regression model was used for adjusting for sex and age. We also conducted stratified analysis by sex and age group using this model. The *χ*^2^ test and the Cochran-Mantel-Haenzel test were used for simple and stratified analyses of knowledge of oral health among PD patients, respectively.

RESULTS: In the present survey, we found the following results. (1) PD patients had more complaints of chewing difficulties and denture discomfort than controls. (2) Fewer PD patients had their own teeth than controls regardless of sex. (3) Fewer PD patients cleaned their dentures every day than controls, regardless of sex or age. (4) More than half of the PD patients had problems with swallowing.

CONCLUSION: We found that PD patients had more complaints about their oral health and more problems in oral health behavior than the general population. These findings may provide useful information for the caregivers of PD patients to conduct oral health care as well as for making oral health plans for PD patients and for medical and welfare services.

There is no established medical treatment to completely cure intractable diseases (i.e., Nanbyo in Japanese) because their etiologies remain unknown. Thus, patients with intractable diseases may suffer from disabilities even after treatment. As the clinical course is chronic, patients with intractable diseases need long-lasting medical treatment and care, which causes a heavy burden for the patients themselves as well as their family members, not only mentally but also financially.

Patients with Parkinson’s disease (PD), which is one of the most common intractable diseases, suffer from disabilities of walking, eating, biting, swallowing, using the toilet, communicating, or respiration through muscle weakness, or disability of movement, in addition to abnormal eye symptoms and autonomic nervous system disorders.^1^^,^^2^ Patients with dysphagia, dental diseases and/or poor oral hygiene have been shown to have a high incidence of aspiration pneumonia,^3^^-^^8^ which may sometimes lead to death,^5^^,^^9^^-^^11^ Therefore, it is important for patients with neurogenic disorders to maintain good oral hygiene.

However, their oral health conditions and related factors, such as oral complains, tooth brushing, condition of swallowing, knowledge of oral health, frequency of having a checkup in a dental clinic, and so on, have not been clearly elucidated.

To the best of our knowledge, only a few surveys of oral health conditions in PD patients have been reported, and only the small numbers of PD patients were surveyed in these studies.^12^^-^^14^ Persson et al.^12^ compared only 30 PD patients with controls, while Nilsson et al.^13^ compared 75 PD patients with controls. However, they did not investigate the oral health conditions, but only the swallowing situation.

Patients with dyskinesia of the hands and/or face often suffer from poor oral hygiene. Patients with PD may be a high-risk group for caries and periodontal disease, and may have more complains of poor oral health and more health problems in the oral cavity than the general population.

The aim of the present study was to investigate oral health conditions and related factors that may influence oral health among patients with PD.

## METHODS

### Subjects

In Japan, patients with PD can receive public financial aid from the government if their disease stage according to Hoehn and Yahr is from III to V,^15^ and the eligible study cases were all of 240 patients with PD who received public financial aid in the Okhotsk area, in 2000. Okhotsk is located in the eastern part of Hokkaido, and is one of tertiary medical-care zones in Hokkaido. Among them, 201 PD patients participated in a meeting held in the city of Abashiri or were certified in the cities of Kitami and Monbetsu. They were asked to take part in this survey, and 109 patients responded (response rate, 54.2%). We used 104 patients aged over 60 years as cases. In 2001, 422 persons got dental check-ups in basic health examinations of cities and towns in Okhotsk. We selected 191 persons aged over 60 years as controls. Sex and age distribution of the 104 cases with PD and 191 controls are shown in Table 1.

**Table 1.** Sex and age distribution of 104 Parkinson’s disease patients and 191 controls.

	Parkinson’s disease			control		
	
	male	female	total	male	female	total
age (year)						
60-69	14	24	38	47	74	121
70-79	25	24	49	28	32	60
80+	5	12	17	3	7	10
total	44	60	104	78	113	191

### Method of Survey

During the period spanning January through March in 2000, we investigated the patients with PD mostly by mail (196 patients), and in part, by interview (5 patients). A structured questionnaire was employed for both patients and controls. As shown in Appendix, common questions in the survey for patients and controls were about oral complains, the presence of their own teeth, tooth brushing, denture condition, and the presence of a family dentist. Question items for all cases and controls were bad breath, swollen gums, chewing difficulties, the presence of their own teeth, and the presence of a family dentist. Those for people with dentures were denture discomfort and denture condition. People having own teeth were asked about toothache, gingival bleeding, food impaction, tooth movement, and tooth brushing. In addition, disability in brushing, the condition of swallowing, having a checkup in a dental clinic, and knowledge of oral health were surveyed among PD patients. A dentist in the Kitami Public Health Center of Hokkaido (the first author) examined the teeth of the 422 controls, and they completed the self-administrated questionnaires by themselves. No interview survey or mailing survey was employed for the controls. The present study was approved by the Ethics Committee of Sapporo Medical University.

### Analyses

We compared the case group with the control group using the unconditional logistic regression model^17^ adjusted for sex and age. The adjusted odds ratio (OR) and its 95% confidence interval (CI) were estimated. We also conducted stratified analysis by sex and age group using this model. The *χ*^2^ test and the Cochran-Mantel-Haenzel test were used for simple and stratified analyses of knowledge of oral health among PD patients, respectively. Tests of statistical significance were based on two-sided P values, and the *α*-error was set at the 5% level. The SAS^®^ system (ver. 8) was employed for the analysis.^17^

## RESULTS

### Results from a case-control study

Table 2 shows results using the unconditional logistic regression model, in men and women after adjusting for age, and in young elderly (from 60 to 69 years old) and old elderly (over 70 years old) after adjusting for age and sex.

**Table 2.** Results of logistic regression analysis using 104 Parkinson’s disease patients and 191 controls.

	total			adjusted for sex, age			by sex adjusted for age						by age strata adjusted for sex, age					
							male			female			from 60 to 69 years old			over 70 years old		

	cases	controls	nature of population	cases	controls	odds ratio	cases	controls	odds ratio	cases	controls	odds ratio	cases	controls	odds ratio	cases	controls	odds ratio
				n (%)	n (%)	(95% CI)	n (%)	n (%)	(95% CI)	n (%)	n (%)	(95% CI)	n (%)	n (%)	(95% CI)	n (%)	n (%)	(95% CI)
Oral complaints																		
① Toothache	60	169	Having own teeth	9 (15)	16 (9)	1.7 (0.7-4.3)	5 (17)	7 (10)	2.0 (0.6-7.2)	4 (13)	9 (9)	1.4 (0.4-5.4)	3 (10)	12 (11)	1.0 (0.3-4.1)	6 (19)	4 (7)	3.9 (0.9-17.6)
② Gingival bleeding	60	169	Having own teeth	8 (13)	21 (12)	1.1 (0.5-2.8)	4 (14)	9 (12)	1.1 (0.3-3.8)	4 (13)	12 (13)	1.2 (0.4-4.3)	5 (17)	16 (14)	1.2 (0.4-3.6)	3 (10)	5 (9)	0.9 (0.2-4.2)
③ Food impaction	60	169	Having own teeth	36 (60)	71 (42)	2.1 (1.1-3.9)	18 (62)	31 (42)	1.8 (0.8-4.5)	18 (58)	40 (42)	2.4 (0.9-5.6)	19 (66)	48 (42)	3.0 (1.2-7.1)	17 (55)	23 (42)	1.5 (0.6-3.8)
④ Bad breath	104	191	All	16 (15)	23 (12)	1.4 (0.7-2.9)	8 (18)	11 (14)	1.6 (0.6-4.3)	8 (13)	12 (11)	1.4 (0.6-3.5)	8 (21)	15 (12)	1.8 (0.7-4.7)	8 (12)	8 (11)	0.9 (0.3-2.9)
⑤ Tooth movement	60	169	Having own teeth	6 (10)	8 (5)	2.0 (0.7-6.3)	4 (14)	4 (5)	2.5 (0.6-11.1)	2 (6)	4 (4)	1.5 (0.3-9.1)	3 (10)	3 (3)	4.7 (0.8-26.5)	3 (10)	5 (10)	1.1 (0.2-5.1)
⑥ Swollen gums	104	191	All	17 (16)	18 (9)	1.8 (0.9-3.9)	6 (14)	7 (9)	1.4 (0.4-4.7)	11 (18)	11 (10)	2.2 (0.9-5.7)	8 (21)	9 (7)	3.4 (1.2-10.1)	9 (14)	9 (13)	1.1 (0.4-2.9)
⑦ Chewing difficulties	104	191	All	29 (28)	12 (6)	6.0 (2.8-12.8)	13 (32)	2 (3)	14.5 (3.0-69.1)	16 (27)	10 (9)	4.2 (1.7-10.5)	11 (29)	9 (7)	4.5 (1.6-12.3)	18 (27)	3 (4)	8.9 (2.5-32.0)
⑧ Denture discomfort	80	104	Using dentures	32 (40)	17 (16)	3.9 (1.9-8.0)	7 (23)	7 (17)	1.7 (0.5-5.6)	25 (51)	10 (16)	6.4 (2.5-16.5)	10 (36)	11 (21)	2.9 (1.0-8.5)	22 (42)	6 (12)	5.0 (1.8-13.9)

No own teeth	104	191	All	38 (37)	22 (12)	3.5 (1.8-6.8)	10 (25)	5 (6)	3.6 (1.0-12.0)	28 (48)	17 (15)	3.5 (1.6-7.7)	6 (17)	7 (6)	3.1 (0.9-10.3)	32 (51)	15 (21)	4.1 (1.8-9.3)
Do not brush teeth every day	60	169	Having own teeth	17 (28)	0 (0)	P < 0.01*	7 (24)	0 (0)	P < 0.01*	10 (32)	0 (0)	P < 0.01*	5 (18)	0 (0)	P < 0.01*	12 (38)	0 (0)	P < 0.01*
Do not clean dentures every day	80	104	Using dentures	19 (24)	3 (3)	10.5 (2.9-37.7)	8 (29)	2 (5)	7.8 (1.4-42.7)	11 (25)	1 (2)	19.4 (2.3-162.9)	5 (21)	1 (2)	13.2 (1.4-121.6)	14 (30)	2 (4)	9.7 (2.0-46.7)
Do not remove dentures and put them in a cup of water	80	104	Using dentures	33 (41)	35 (34)	1.8 (1.0-3.5)	15 (48)	12 (29)	2.4 (0.9-6.4)	18 (38)	23 (37)	1.6 (0.7-3.8)	17 (63)	31 (58)	2.4 (0.9-6.4)	16 (31)	13 (25)	1.4 (0.6-3.5)
Do not have own family dentist	104	191	All	24 (23)	54 (28)	0.8 (0.4-1.4)	7 (18)	23 (29)	0.6 (0.2-1.5)	17 (30)	31 (27)	0.9 (0.4-2.0)	7 (21)	33 (100)	0.7 (0.3-1.8)	17 (28)	21 (30)	0.8 (0.4-1.8)

CI: confidence interval

—: not calculated

*: Fisher’s exact test

As shown in Table 2, more PD patients complained of chewing difficulties (OR = 6.0, 95% CI:2.8-12.8) after adjustment for sex and age. After adjusting for age, more PD patients complained of them than controls among both men (OR = 14.5, 95% CI:3.0-69.1) and women (OR = 4.2, 95% CI:1.7-10.5). In addition, more PD patients complained of chewing difficulties (young: OR = 4.5, 95% CI:1.6-12.3; old: OR = 8.9, 95% CI:2.5-32.0) than controls in both young and old elderly.

Persons without their own teeth were more commonly seen among the PD patients than in the control group adjusted for sex and age (OR = 3.5, 95%CI:1.8-6.8). PD patients more commonly lacked their own teeth than controls in both sexes after adjusting for age (men: OR = 3.6, 95% CI:1.0-12.0; women: OR = 3.5, 95% CI:1.6-7.7). Among the old elderly, those without their own teeth were more commonly seen among PD patients than controls after adjusting for age and sex (OR = 4.1, 95% CI:1.8-9.3).

Among the young elderly, more PD patients complained of swollen gums than controls after adjusting for age and sex.

There were no differences between the two groups about having their own family dentists. Of the PD patients, 71 (68%) had their own family dentists.

More PD patients complained of denture discomfort (OR = 3.9, 95% CI:1.9-8.0) than controls after adjustment for sex and age. Although the proportion of those who complained of denture discomfort did not differ between the two groups for men, more female PD patients complained of denture discomfort than female controls. More PD patients complained of denture discomfort (young: OR = 2.9, 95% CI:1.0-8.5; old: OR = 5.0, 95% CI:1.8-13.9) than controls in both the young and old elderly.

Fewer PD patients cleaned their dentures every day than in the control group after adjustment for sex and age (OR = 10.5, 95% CI:2.9-37.3). Fewer PD patients cleaned their own dentures every day than controls in both sexes. Fewer PD patients cleaned their dentures every day than controls in both the young and old elderly after adjusting for age and sex.

There were no differences between the PD patients and the control group about storing their own dentures correctly before sleeping. Forty-five PD patients (56%) removed their own dentures and put them in a cup of water before sleeping.

More PD patients complained of food impaction than controls after adjustment for sex and age. Among the young elderly, more PD patients complained of food impaction than controls after adjusting for age.

Fewer PD patients brushed their teeth every day than in the control group (p<0.01), in both sexes (p<0.01), and in both the young and old elderly (p<0.01).

### Knowledge and condition of oral health among PD patients

Few PD patients had knowledge of dental floss (3%), brushes for cleaning dentures (19%) and coating of the tongue (14%).

Table 3 shows oral-health conditions among 104 PD patients. Fifty-one PD patients (49%) had trouble brushing their teeth or cleaning their dentures, 38 patients (37%) had difficulty gargling. As for swallowing, 56 PD patients (54%) answered that they had some problems. Among the 60 patients who had their own teeth, 21 (35%) had trouble brushing by themselves. Among 80 PD patients who had dentures, 10 (13%) had some problems about either removing or putting them in by themselves. Concerning regular checkups of their dentures, 24 PD patients (30%) had never gone to a dental clinic after they had been made. Seven patients (7%) had been refused treatment in a dental clinic due to their disease. Fifty patients (48%) answered that they were able to go to a dental clinic if there was assistance by either family members or helpers, and transportation service by car, and 10 (10%) could not go to a dental clinic in any case. Of these 10 patients, 9 (90%) wanted to use home-visiting dental services. As for the content of home-visiting dental service, 62 patients (60%) wanted to have a dental check-up and tooth brushing instruction either at present or in the future.

**Table 3.** Conditions of oral health among 104 Parkinson’s disease patients.

○	Disability in brushing their teeth or cleaning their dentures	
	Number having trouble	51 (49%)
	Number having no trouble	45 (43%)
	Number of nonresponders	8 (8%)
	Total	104 (100%)

○	gargling	
	Number able to gargle	63 (60%)
	Number only able to put a single swallow of water in their mouth	30 (29%)
	Number not able to gargle at all	8 (8%)
	Number of nonresponders	3 (3%)
	Total	104 (100%)

○	Swallowing	
	Number with no problem	41 (39%)
	Number always choking during eating or drinking	12 (12%)
	Number choking only when they drank a cup of water	5 (5%)
	Number sometimes choking	39 (37%)
	Number of nonresponders	7 (7%)
	Total	104 (100%)

○	Brushing	
	Number able to brush their teeth by themselves	39 (65%)
	Number able to brush their teeth with assistance	5 (8%)
	Number not able to brush their teeth by themselves	8 (13%)
	Number of nonresponders	8 (13%)
	Total (having their own teeth)	60 (100%)

○	Denture wearing	
	Number removing and putting their dentures in by themselves	66 (82%)
	Number either removing or putting their dentures in by themselves	3 (4%)
	Number neither removing nor putting their dentures in by themselves	7 (9%)
	Number of nonresponders	4 (5%)
	Total(having own dentures)	80 (100%)

○	Access to a dental clinic	
	Number able to go to a dental clinic alone	31 (30%)
	Number able to go to a dental clinic if there was assistance by family members	30 (29%)
	Number able to go to a dental clinic if there was assistance by helpers	5 (5%)
	Number not able to go to a dental clinic without transportation service by car	15 (14%)
	Number not able to go to a dental clinic in any case	10 (10%)
	Number of nonresponders	13 (12%)
	Total	104 (100%)

○	Content of home-visiting dental service	
	Number looking forward to have a dental check-up and tooth brushing instruction	28 (27%)
	Number not needing them at that time but wanted to use a home-visiting dental service in the future	34 (33%)
	Number not looking forward to home-visiting dental service	25 (24%)
	Number of nonresponders	17 (16%)
	Total	104 (100%)

## DISCUSSION

As far as we know, this is the first study of oral health conditions in PD patients compared with controls in Japan. Although Fukayo^14^ reported about oral health conditions of PD patients, they did not compare them with controls.

### The oral health conditions of 104 PD patients

In the present study, those who complained of chewing difficulties were more common among PD patients than controls after adjustment for sex and age. This indicated that chewing difficulties might be common in PD patients. This may be explained by the following reasons. First, PD patients may suffering from dyskinesia such as flycatcher tongue and lip.^18^ Second, PD patients may be suffering from mastication disorders with oral dyskinegia^7^^,^^19^ or xerostomia^20^ caused by the administration of anticholinergics and so on. In contrast, Persson et al.^12^ failed to show a significant difference in the rate of those with chewing difficulties between PD patients and a control population sample because of the small sample size (10 of 30 PD patients [33%] complained of chewing difficulties, as did 20 of 526 controls [4%]).

The present study revealed that more PD patients complained of denture discomfort, in particular female patients. This result was consistent with the report by Kieser et al.^21^ suggesting that a third of PD patients have loose dentures or poor denture control. This may be explained by the following reasons. First, PD patients may be suffering from oral dyskinesia^7^^,^^19^ or xerostomia^20^ caused by the use of anticholinergics. Second, PD patients may be suffering from lack of muscle coordination and rigid facial muscles that jeopardize denture retention and control^20^. Many female, but not so many male, PD patients complained of denture discomfort. It is possible that, compared with male PD patients, female PD patients might suffer from denture discomfort caused by involuntary movements of some facial muscles, the tongue and lips, as oral dyskinesia is more commonly seen in elderly woman than in elderly men^4^^,^^7^.

The present study showed that many PD patients did not have their own teeth, regardless of sex. This result was the opposite of the results of studies reported in Europe showing that caries were generally less common in PD patients than in controls, and that teeth were retained longer in PD patients than in controls.^12^^,^^18^ This may be explained by the following possibilities. Many PD patients in the present survey had lost their teeth due to many years of poor oral hygiene because fewer PD patients brushed their teeth every day or they had difficulty going to a dental clinic in the early stage of caries. The results of the present study may suggest that the support for oral health for PD patients in Japan remains less sufficient than in Europe.

Because PD patients often suffer from heartburn and nausea, causing a decrease in oral hygiene^6^^,^^21^ as well as xerostomia, leading to an increased risk of caries due to the use of anticholinergics or monoamine oxidase inhibitors,^18^^-^^21^ PD patients may have a high risk of losing their teeth.

### Swallowing among PD patients

Fifty-six of the 104 (54%) PD patients had some subjective problems swallowing. In western countries^6^^,^^20^^,^^22^ about half of PD patients are reported to have dysphagia. However, we may have underestimated the proportion of the PD patients with dysphagia in the present study because of the following reasons. First, several studies have revealed that PD patients have swallowing difficulties without any subjective symptoms.^5^^,^^11^^,^^13^^,^^23^ Second, Nilsson et al.^13^ found dysphagia in more than 90% of PD patients who were in the same stages as our study subjects (Hoehn and Yahr stages III and IV). Most of the PD patients in the present survey may have had poor oral hygiene, because only a few of them brushed their teeth every day. Therefore, they might be susceptible to aspiration pneumonia via aspiration of saburra and indigenous oral bacterial flora.^5^^,^^6^^,^^11^^,^^23^ In addition, most elderly PD patients may have a high risk of severe aspiration pneumonia because elderly people are more likely to contract oral candidiasis^7^^,^^8^ and fatal moniliasis pneumonia^8^ when they have poor oral hygiene and denture discomfort.

Bucbboz^24^ reported that the most common cause of death among progressive PD patients was aspiration pneumonia.

### Oral health behavior of PD patients

We found that very few PD patients brushed their teeth every day or cleaned their dentures every day. They probably had difficulties because of their PD symptoms such as resting tremors, akinesia and bradykinesia.^20^^-^^22^^,^^25^^,^^26^

Even though most PD patients complained of food impaction, few PD patients had knowledge about the interdental brush. Therefore, it is very important for PD patients to get appropriate dental health advice from a dentist or dental hygienist, especially from their own family dentists. This is consistent with the report by Kieser et al.^21^ who recommended that patients with neurode-generative disorders should be followed by the same dentist.

However, most PD patients wanted to have dental treatment and dental health services by home visits because it was very difficult for them to receive those services at dental clinics unless their families took them there. Fiske et al.^20^ reported that domiciliary dental care was important for PD patients because one of the major barriers to receive dental care was access to dental premises.

The results of the present study may show that many kinds of support (e.g., environmental considerations and advice on oral care for families of PD patients and their caregivers, usual support of oral care at the home by a welfare agency such as a home nursing station, special transport service to go to the dental clinic, and an increase of dental clinics doing dental treatment by home visits) are necessary for PD patients to maintain good oral health conditions, to prevent aspiration pneumonia and to have good quality of life.

There are some limitations to our study. First, information on most PD patients was obtained by mail; only 5 PD patients were interviewed. Because information of mailed self-administered questionnaire may be inferior to one by personal interview in quality, there is a possibility of information bias to some extent in our study. Second, we did not check the duration of the disorder. There is a possibility of survival bias, because we used not only incident cases but also prevalent cases. Third, the controls were not randomly selected from the general population, but were recruited from persons at health checkups. The tooth characteristics of 191 controls compared with participants of a survey on dental diseases in 1999^16^ are shown in Table 4. Averages of tooth characteristics in the control group were calculated by adjusting age with a direct method using the data of the survey on dental diseases in 1999^16^ as a standard population. As shown in Table 4, more controls had their own teeth than in the general population among males. We must consider the effect of using healthy participants as controls.

**Table 4.** Tooth characteristics of 191 controls compared with general population.^16^

	average number of intact teeth	average number of treated teeth	average number of untreated teeth	average number of missing teeth	average number of DMFT*	proportion of people with edentulous jaw (%)
	Male					
Control group adjusted for age^†^	9.1	8.8	0.8	9.4	18.9	6.4
General population (over 60 years old )	7.3	7.7	1.4	12.1	21.2	14.3

	Female					
Control group adjusted for age^†^	4.7	10.9	0.3	12.1	23.3	15.0
General population (over 60 years old )	5.3	8.0	1.0	13.9	22.9	17.1

*: Average number of decayed teeth, missing teeth or filled teeth per person.

^†^: Averages of tooth characteristics in the control group were calculated by adjusting age via a direct method using the data of the survey on dental diseases in 1999^16^ as a standard population.

In conclusion, we found that PD patients had more complaints about their oral health (e.g., chewing difficulties, denture discomfort) and more problems in the oral health behavior than the general population. In addition, more than half of PD patients had problems with swallowing. These findings may be useful for the caregivers of PD patients to conduct oral care as well as for making health plans for dental care for PD patients and for medical and welfare services. Furthermore, dental staff members in public health centers should strengthen training for caregivers to enhance knowledge and skills about oral care, as well as dental checkups and dental health services by home visits for PD patients.

## Appendix. Questionnaire for the survey.

**Table:**

○	What are your oral complaints ? Please check all which apply.		
	① Toothache	② Gingival bleeding	③ Food impaction
	④ Bad breath	⑤ Tooth movement	⑥ Swollen gum
	⑦ Chewing difficulties	⑧ Denture discomfort	

○	Do you have your own teeth (including capped teeth) ?		
	① yes	② no	

○	Do you brush your teeth every day?		
	① yes	② sometimes or never	

○	Do you clean your dentures every day?		
	① yes	② sometimes or never	

○	Where do you keep your dentures while sleeping?		
	① remove dentures and put them in the cup of water		
	② remove dentures and leave them in air, or keep them in your mouth		

○	Do you have your own family dentist?		
	① yes	② no	
